# The Effect of Enhanced External Counterpulsation (EECP) on Quality of life in Patient with Coronary Artery Disease not Amenable to PCI or CABG

**DOI:** 10.7759/cureus.7987

**Published:** 2020-05-06

**Authors:** Rafiullah Jan, Asghar Khan, Salman Zahid, Abdul Sami, Syed M Owais, Fahad Khan, Sayyed Jalawan Asjad, Muhammad Hassan Jan, Zahid A Awan

**Affiliations:** 1 Cardiology, Hayatabad Medical Complex, Peshawar, PAK; 2 Internal Medicine, Khyber Teaching Hospital, Peshawar, PAK; 3 Cardiology, Hayatabad Medical Complex Peshawar, Peshawar, PAK; 4 Internal Medicine, Hayatabad Medical Complex, Peshawar, PAK

**Keywords:** coronary artery disease, quality of life, heart failure, counterpulsation

## Abstract

Enhanced External Counterpulsation (EECP) is a non-invasive FDA approved therapy for patients with refractory angina pectoris. The EECP mechanism of action is similar to that of an intra-aortic balloon pump (IABP) by administering a vigorous pressure pulse via external blood pressure cuffs during the diastole. The benefit of EECP includes improvement in angina severity, angina stability, maximal walking capacity and generalized improvement in overall health. Seatle Angina Questionnaire (SAQ) is a valid, reliable and sensitive measure of health-related quality of life. It is also a sensitive and reproducible evaluation tool to measure the response to an intervention. We did a pre-test post-test designed prospective study to evaluate the effect of EECP on the quality of life in patients with CAD. There was a significant difference between SAQ-7 health-related quality of life between the pre-EECP and post-EECP groups (P value= <0.01). Moreover, a positive correlation was reported between the New York Heart Association (NYHA) Functional Classification before treatment and post-EECP SAQ-7 health-related quality of life (P value=0.015).

## Introduction

Enhanced External Counterpulsation (EECP) is a non-invasive FDA approved therapy for patients with refractory angina pectoris [1]. A significant number of patients are not fit for either coronary artery bypass graft surgery (CABG) or revascularization. According to one estimate, 2.4 million people in the US with CAD are not suitable candidates for bypass or PCI. In such patients, EECP can prove to be an effective alternative [2].

The EECP mechanism of action is similar to that of an intra-aortic balloon pump (IABP) by administering a vigorous pressure pulse via external blood pressure cuffs during the diastole. This translates into increased coronary blood flow during the diastole to the coronary arteries which has been proven with the help of Doppler and angiographic methods [3]. Moreover, it has been postulated that EECP can stimulate the formation of new collateral vessels that can ultimately translate into long term symptomatic improvement of angina symptoms [4]. 

EECP has proven to be a safe, effective and non-invasive therapy for a patient with refractory angina. It has been shown to improve the health-related quality of life in diabetic and non-diabetic patients with CAD. The benefit of EECP includes improvement in angina severity, angina stability, maximal walking capacity and generalized improvement in overall health [5]. According to one study, the heart benefits on quality of life were shown to have been sustained for a period of three years [6].

Seatle Angina Questionnaire (SAQ) is a valid, reliable and sensitive measure of health-related quality of life. It is also a sensitive and reproducible evaluation tool to measure the response to intervention [7]. SAQ includes five categories, namely Physical Limitation, Angina Stability, Angina frequency, treatment satisfaction and quality of life. Scores for each category are scaled from 0 to 100 with 0 being the worst performance and 100 being the best performance status. We used a smaller version of SAQ questionnaire called SAQ-7 [8]. 

The study aims to show the effectiveness of EECP on quality of life in patients with coronary artery disease not amenable to PCI or CABG in a tertiary care hospital of Peshawar, Pakistan. 

## Materials and methods

A pre-test post-test designed prospective study was carried out in the Department of Cardiology, Hayatabad Medical Complex, Peshawar, Pakistan. The study duration was from January 2014 to May 2019. Two hundred and twenty patients were included in the study using consecutive sampling technique. 

A cardiac team meeting comprising of a Cardiac surgeon, Cardiac Anesthesiologist, and a panel of Interventional cardiologists discussed the cases that qualified for EECP therapy.

Patients who had CAD with angina or SOB, Post CABG patients with angina, an angiographically critical disease not amenable to PCI or CABG and the patients who were unwilling to undergo PCI or CABG were included. Patients belonging to all gender and age groups were included in the study. 

Patients with Peripheral arterial disease (PAD), significant Aortic Regurgitation (AR), uncontrolled hypertension and aortic aneurysms were excluded from the study. Patient’s who refused to give consent were also excluded from the study. 

Two-hundred and twenty patients were recruited for the study. Before administering EECP treatment, SAQ-7 was used to assess the health-related quality of life along with the baseline characteristics of the patient. A total of thirty-five sessions of EECP were carried out in the morning and evening. Each session of EECP lasted one hour. At three-months follow-up after EECP treatment, health-related quality of life of the patient was assessed using the SAQ-7. 

SPSS, Version 22 (IBM Corp., Armonk, NY, US) was used to enter and analyze the data. An analysis of the comparison between pre-treatment and post-treatment group was performed to compare SAQ health-related quality of life and p-value was calculated. A Spearman correlation was performed to see the correlation between heart failure severity and improvement in health-related quality of life Post-EECP. Data was presented in the form of tables, bar charts and scatter plot. 

## Results

The mean age of the patients was 64. Out of 220 patients, 103 (46.8%) were male patients and 117 (53.1%) were female patients. With regards to diabetes, 100 (45.5%) patients were diabetic. Hypertension as a comorbidity was present in 122 (55.5%) patients while 136 (61.8%) patients HAD a history of myocardial infarction. Twenty five patients (11.4%) had undergone CABG. A left ventricular ejection fraction (EF) between 35-45 was present in 77 (35%) patients. An EF of 45-50 in 40 (18.2%) patients and EF>50 in 103 (46.8%) patients was observed. Coronary angiography revealed that Double vessel coronary artery disease was present in 47 (21.4%) patients and triple vessel coronary artery disease was present in 173 (78.6) patients. Patients' severity of heart failure was categorized by using Newyork Heart Association (NYHA) classification. Out of 220 patients, 56 (25.5%) had NYHA IV, 132 (60%) had NYHA III, 31 (14.1%) had NYHA II and only 1 (0.5%) was classified as NYHA I. This data is summarized in Table 1. 

**Table 1:** Baseline characteristics of patients. EF: Ejection fraction, CABG: Coronary artery bypass graft surgery, DVCAD: Double vessel coronary artery disease, TVCAD:Tripple vessel coronary artery disease, NYHA: Newyork Heart Assoication Classification for heart failure

Variable	N	Percentage
Male patients	103	46.8%
Female Patients	117	53.1%
Diabetics	100	45.5%
Non-Diabetics	120	54.5%
Hypertensive	122	55.5%
Non-Hypertensive	98	44.5%
History of myocardial infarction	136	61.8%
No history of Myocardial infarction	84	38.2%
Status post CABG	24	11.4%
EF>50	103	46.8%
EF 45-50	40	18.2%
EF 35-45	77	35%
DVCAD	47	21.4%
TVCAD	173	78.6%
NYHA Class IV	56	25.5%
NYHA Class III	132	60%
NYHA Class II	31	14.1%
NYHA Class I	1	0.5%

**Figure 1:**
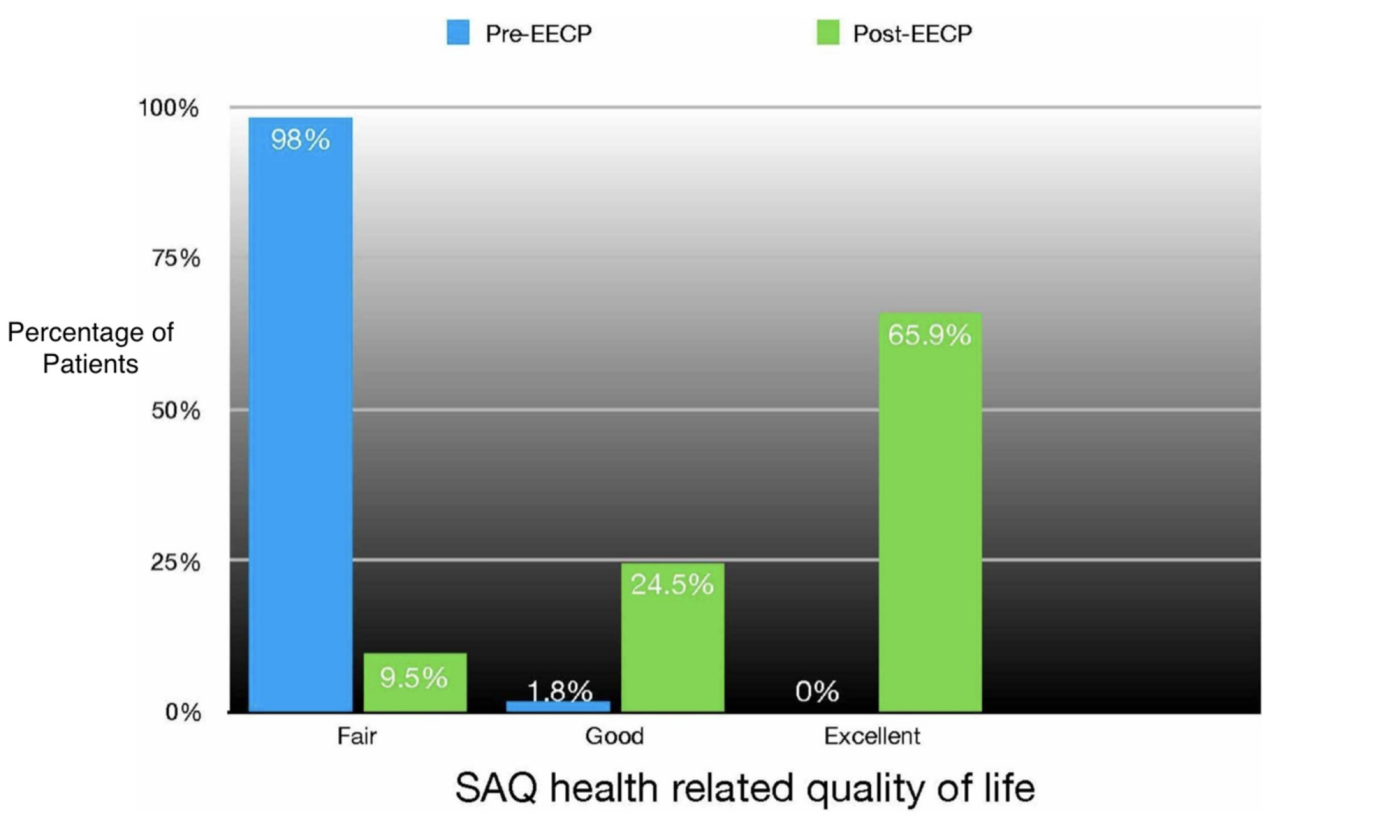
Pre-EECP versus Post-EECP comparision of health realted quality of life EECP: Enhanced External Counterpulsation, SAQ: Seatle Angina Questionnaire, Fair: SAQ-7 score (0-<50), Good: SAQ-7 score (50-75) , Excellent: SAQ-7 score (75-100)

The pre-EECP SAQ-7 questionnaire showed a “fair” quality of life in 98.2 % of patients and 1.8% of patients were categorized as “good”. The post-EECP SAQ-7 questionnaire showed marked improvement in the quality of life with 65.9% of patients categorized as “excellent”, 24.5% of patients were classified as “good” and only 9.5% of patients classified as “fair”. P value was calculated which showed a significant difference between the SAQ-7 health related quality of life between the pre-EECP and post EECP groups with p value<0.05 (Table 2)(Figure1).

**Table 2:** Pre-EECP versus Post-EECP comparison of health related quality of life. EECP: Enhanced External Counterpulsation, SAQ: Seatle Angina Questionnaire, Fair: SAQ-7 score (0-<50), Good: SAQ-7 score (50-75) and Excellent: SAQ-7 score (75-100)

SAQ	Pre-EECP	Post-EECP	P value
Fair	98.2%	9.5%	<0.05
Good	1.8%	24.5%	
Excellent	0%	65.9%	

**Figure 2:**
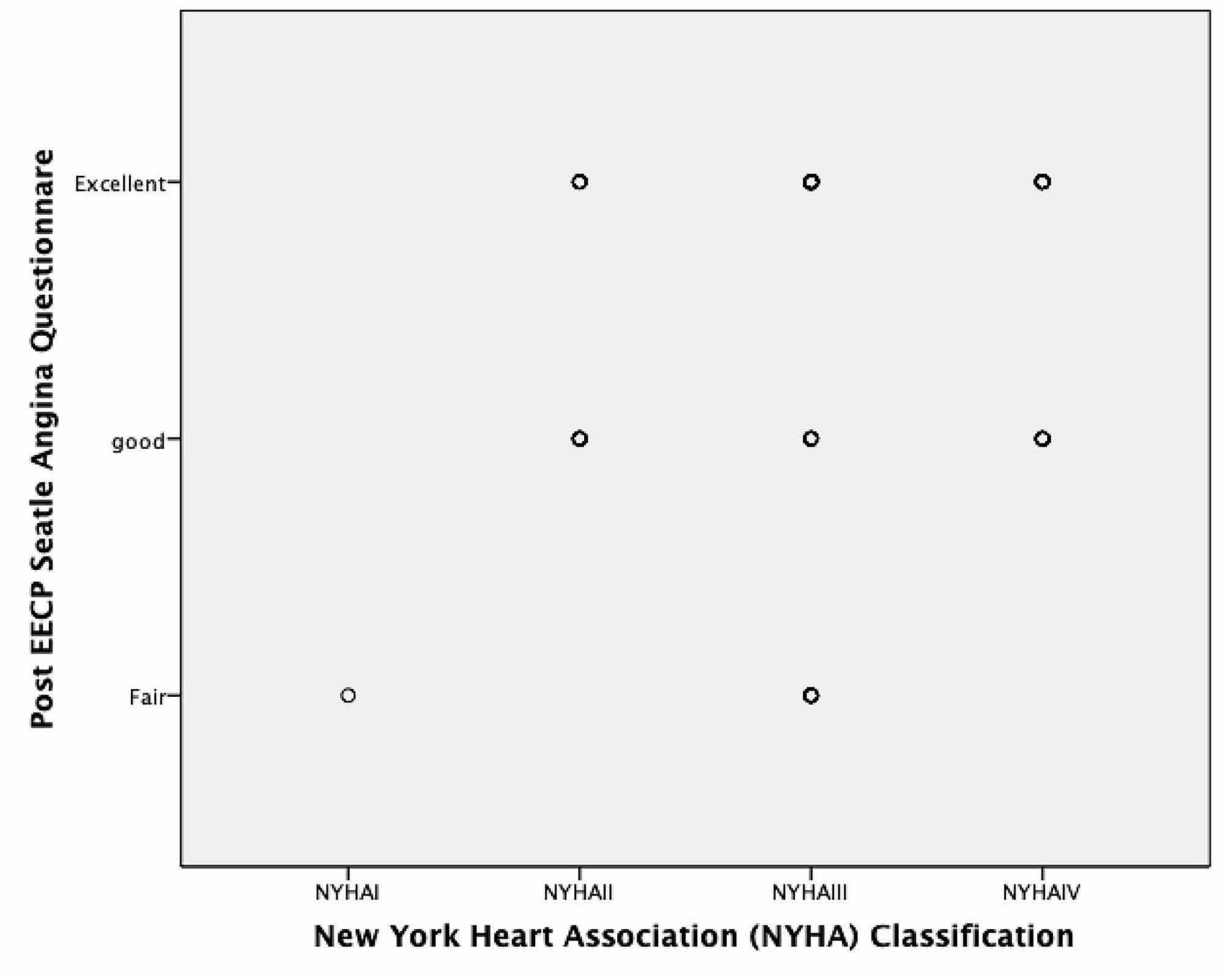
Correlation between Post EECP SAQ score with NYHA Classification EECP: Enhanced External Counterpulsation, SAQ: Seatle Angina Questionnaire, Fair: SAQ-7 score (0-<50), Good: SAQ-7score (50-75) , Excellent: SAQ-7 score (75-100)

A statistically signifiant positive correlation was found between severity of symptoms as classified by NYHA classification before administering EECP and post-EECP SAQ-7 score (Corelation co-efficent=0.164 and p value=0.015) (Figure 2).

## Discussion

Our study showed significant improvement in the quality of life following treatment with EECP. The findings of our study are supported by a previous study done on the topic [9]. Moreover, we report a significant positive correlation between NYHA classification before the institution of treatment and post-EECP SAQ-7 score. In another study done on the topic, the PEECH trial demonstrated that 31.3% of the patients showed improvement in NYHA classification 6-months after treatment with EECP [10].

The Seattle Angina Questionnaire is a very sensitive, valid and reproducible instrument for coronary artery disease assessment and is very responsive to intervention. Its components are physical limitation, angina stability, angina frequency, treatment satisfaction and quality of life. The range for each of these components is scored from 1-6. The scale is then devised with 0 being the worst and 100 being the best possible state [7]. SAQ has been the instrument of choice to measure the quality of care in patients with CAD and also to assess outcomes in clinical trials [11]. However, the use of SAQ is limited due to a large number of questions (19 questions) and the lack of a final summary score that can give an idea about the overall assessment of the patient’s health [7]. Chan et al tried to solve the problem by devising a shorter version of SAQ called SAQ-7. They selected seven items from the original SAQ that demonstrated the highest concordance with the original questionnaire. The shorter version of the questionnaire preserves the sensitivity and validity of the original one and also is easier to use in routine care and clinical trials. In SAQ-7, the scale of 0-100 was classified into three groups Fair (0-<50), Good (50-75) and Excellent (75-100) [8]. We used the shorter version SAQ -7 and classified the outcome score into ordinary variables of fair, good and excellent. 

A previous study has shown that EECP has a significant beneficial effect on the exercise capacity and prolongs the patient’s ability to exercise for a longer period of time. In addition to these improvements, the peak increase in oxygen consumption remains unchanged which resulted into fewer angina episodes. [10]. Another study demonstrated that at 12-months follow-up, patients reported a significant improvement in the quality of life as measured by SF-35 and cardiac version of the Quality of Life Index. Moreover, the beneficial effects of EECP were found similar to that of vigorous exercise, where collateral vessels are formed to improve coronary perfusion [12]. Two studies done on a similar topic reported that the beneficial effects of EECP were sustained for a period of two years. Fifty percent of the patients reported a significant improvement in their quality of life after EECP at two years to follow up [13]. A study done on 364 patients reported a 72% improvement in the severity of angina from severe angina to no or mild angina along with a significant improvement in the quality of life after receiving EECP at 2 years of follow-up [14]. Yet another case series by Ahmed et al done in Islamabad, Pakistan showed statistically significant improvement in the mean 6 min walk test after administration of EECP therapy in patients with refractory angina [15].

A randomized controlled trial done on 187 patients with NYHA class II or III symptoms were randomized into EECP plus optimal medical therapy and optical medical therapy alone. One of the secondary endpoints was the mean change in NYHA classification and quality of life. NYHA classification significantly improved in the EECP GROUP at 1 week, 3 months and 6 months duration. However, the improvement of NYHA classification was not demonstrated to persist in the long term [16]. In our study, follow-up after treatment with EECP was three months. 

Currently, there is Class IIb evidence supporting the use of EECP for relief of refractory angina in patients with stable ischemic heart disease [17]. The existing data which is mostly from uncontrolled studies supports benefit in angina refractory to therapy. However, additional data from well-designed RCTs are needed to clarify the therapeutic role of EECP and to make a Class 1A recommendation [18].

## Conclusions

Enhanced External Counterpulsation (EECP) is a non-invasive alternative therapy for patients who are not fit for CABG or PCI. EECP has been shown to be an effective treatment strategy that can improve the quality of life of patients. EECP therapy has a long-lasting effect and can lead to beneficial structural changes in the heart that can improve heart failure symptoms.
